# Four-dimensional computed tomography ventilation imaging-guided radiotherapy planning in different techniques for lung cancer

**DOI:** 10.3389/fonc.2026.1858960

**Published:** 2026-06-01

**Authors:** Shu-Qing Yang, Hui Yu, Yu-Long Yang, Cheng Wang, Yan Li, Di-Feng Guo, Chen-Shi Lin, Chao Li, Zhi-Qiang Cai

**Affiliations:** 1 Department of Oncology, The First Affiliated Hospital of Yangtze University, Jingzhou, China; 2 Department of Radiotherapy Center, Affiliated Cancer Hospital and Institute of Guangzhou Medical University, Guangzhou, China; 3 Department of Orthopedics, Anyang People’s Hospital Affiliated to Henan Medical University, Anyang, China; 4 School of Physics and Optoelectronic Engineering, Yangtze University, Jingzhou, China; 5 Department of Radiotherapy Center, The First Affiliated Hospital of Yangtze University, Jingzhou, China

**Keywords:** 4D-CT ventilation imaging, hybrid IMRT, intensity-modulated radiotherapy (IMRT), lung cancer, radiotherapy, volumetric modulated arc therapy (VMAT)

## Abstract

**Purpose:**

This simulation study aims to integrate four-dimensional computed tomography (4DCT) ventilation imaging into functional planning using different radiotherapy (RT) techniques and compare the dosimetric differences across these techniques.

**Methods:**

4DCT ventilation images from eighteen lung cancer patients were created using Jacobian values obtained via deformable image registration and a ventilation imaging algorithm. For each patient, we designed both anatomical and functional plans using intensity-modulated radiotherapy (IMRT), hybrid IMRT (static plus IMRT beams treated concurrently), and volumetric modulated arc therapy (VMAT). Dosimetric parameters were systematically compared, with particular attention to the functional lung (FL) radiation dose.

**Results:**

The results showed that regardless of the RT technique (IMRT, hybrid IMRT, or VMAT), functional planning reduced fV_5_ (fVx: percentage of functional lung volume receiving ≥ x Gy), fV_10_, fV_20_, fV_30_, and functional mean lung dose (fMLD), while maintaining planning target volume (PTV) dosimetric coverage. Although functional planning increased the radiation dose to most organs at risk (OARs), these increases were not statistically significant. Among the different RT techniques, compared with f-hybrid IMRT, both f-IMRT and f-VMAT reduced radiation dose to the FL and OARs, while achieving superior PTV conformity. Further comparison between f-IMRT and f-VMAT showed that f-VMAT resulted in a lower mean dose (D_mean_) to the esophagus.

**Conclusion:**

Compared with anatomical planning, functional planning reduces the radiation dose to the FL while preserving target dose coverage and meeting dose constraints for OARs. Among the RT techniques for functional planning, f-VMAT may serve as the preferred technique.

## 1 Introduction

Lung cancer severely endangers human health. According to data from the GLOBOCAN 2020 research report, lung cancer caused approximately 1.8 million deaths, establishing it as the most common cause of cancer mortality worldwide (1). Radiotherapy (RT), as an indispensable core treatment modality, studies have shown that 77% of lung cancer patients have evidence-based indications for RT during treatment (2). However, while RT kills tumor cells, it inevitably causes damage to normal lung tissue. Despite continuous advancements in RT techniques, the incidence of radiation-induced lung injury (RILI) remains high (3).

Conventional anatomical planning primarily reduces the incidence of RILI by limiting the irradiated volume and dose to lung tissue. Such planning assumes that lung function is uniformly distributed. However, in reality, human lung function exhibits significant heterogeneity (4). By failing to account for this heterogeneity, anatomical planning may cause high-functioning lung tissues to receive unnecessarily high radiation dose. This limitation has driven the development of lung functional imaging techniques, and various acquisition methods are now available, including single-photon emission computed tomography (SPECT), positron emission tomography (PET), magnetic resonance imaging (MRI), and computed tomography (CT) (5, 6). Among these methods, four-dimensional computed tomography (4DCT) stands out due to its advantages of high resolution, no requirement for radioactive tracers, and low cost (7). Multiple existing studies have compared 4DCT ventilation imaging with pulmonary function tests (PFTs), as well as various lung functional imaging modalities including SPECT and PET, demonstrating its favorable reliability and feasibility (8–10).

Previous studies have confirmed that, across various RT techniques including 3-dimensional conformal radiation therapy (3D-CRT), intensity-modulated radiotherapy (IMRT), volumetric modulated arc therapy (VMAT), stereotactic body radiation therapy (SBRT), and intensity modulated proton therapy (IMPT), 4DCT ventilation imaging-guided functional planning could effectively reduce the functional lung (FL) radiation dose while satisfying all clinical dose constraints (11–15). However, the application of hybrid IMRT, a technique integrating static and IMRT fields, has not yet been reported for functional planning. This study is the first to integrate 4DCT ventilation imaging into functional planning using hybrid IMRT and to compare dosimetric differences with those of functional IMRT (f-IMRT) and f-VMAT.

## 2 Materials and methods

### 2.1 Patient characteristics

This study enrolled eighteen patients with pathologically confirmed stage III-IV lung cancer from our hospital who agreed to receive RT between August 2023 and June 2025. All patients had good performance status with a karnofsky performance status (KPS) score ≥ 80 and had complete 4DCT and simulation CT imaging data. Exclusion criteria included prior chest RT or RT to other sites during this course that might interfere with the chest RT planning. Patients with severe respiratory diseases or significant pulmonary imaging abnormalities, such as anatomical pulmonary abnormalities, were also excluded. **Table 1** presents the detailed characteristics of all patients included in this study.

**Table 1:** Patient characteristics (n = 18).

Parameter	Median (range) or n (%)
Age (years)	64 (52-79)
Sex	
Male	15 (83%)
Female	3 (17%)
Diagnosis	
NSCLC	13 (72%)
SCLC	5 (28%)
Stage	
III	9 (50%)
IV	9 (50%)
PTV (cm^3^)	205.27 (44.84-376.72)
Tumour laterality	
Left	6 (33%)
Right	12 (67%)
Tumour location	
Central	14 (78%)
Peripheral	4 (22%)

NSCLC, non-small cell lung cancer; SCLC: small cell lung cancer; PTV, planning target volume.

### 2.2 CT simulation and 4DCT scanning

All patients were placed in a supine position, and their body positions were fixed using an integrated carbon fiber plate combined with a thermoplastic film or a vacuum air cushion. After intravenous contrast administration, patients underwent contrast-enhanced chest CT on a Philips Brilliance 16-slice simulation scanner (Philips Medical Systems, Inc) with 5 mm slice thickness. Subsequently, pressure sensors were used to capture in real time the pressure changes on the patient’s body surface caused by breathing, generating a respiratory signal waveform. During the patient’s free-breathing state, the system synchronously performed 4DCT scanning, divided a single complete respiratory cycle into 10 phases, and then reconstructed CT images corresponding to each phase, thereby enabling dynamic imaging for tracking respiratory motion. Finally, the obtained CT images were transferred to the Varian Eclipse (Varian Medical Systems, USA) treatment planning system (TPS) for subsequent target delineation and treatment planning design.

### 2.3 4DCT ventilation imaging

The 10 respiratory phase images transferred from the 4D-CT workstation to Eclipse were combined to generate an average density projection (AIP) image. The end-inspiratory CT images, end-expiratory CT images, and AIP images were selected, and the whole lung regions were automatically generated on these images using an auto-segmentation system, followed by manual modification. Subsequently, the end-inspiratory (00%) and end-expiratory (50%) images were exported to 3D-Slicer (Version 5.6.2) for image segmentation, with corresponding VTK files generated. The VTK files were imported into the lung ventilation function analysis system (ZHANGShuxu 4D-CT LF, V1.0, copyright registration No. 0396648). Multi-resolution (level 5) 3D B-spline deformable image registration (DIR) was performed on the two images using the embedded Elastix toolbox (Version 4.4), with 256 iterations per level. Mutual information was adopted as the similarity metric, and the spatial transformation parameters were iteratively updated via a parameter optimization algorithm until convergence. After registration, the deformation vector field (DVF) was converted to the Jacobian matrix using the transformation command of Elastix. Finally, the Jacobian matrix data and AIP image files were imported into 3D-Slicer for visualization and quantitative analysis of pulmonary ventilation function. Jacobian > 1 indicates lung tissue expansion, while Jacobian< 1 suggests contraction (16). In this study, to accurately identify lung regions with high ventilation activity and with reference to previous studies (17, 18), regions with a Jacobian determinant value > 1.2 were defined as FL, corresponding to high-ventilation regions. Subsequently, the high-ventilation region distribution map was fused with the planning CT.

### 2.4 Target delineation and dosimetric constraints

The target delineation was performed by senior radiation oncologists in accordance with International Commission on Radiation Units and Measurements (ICRU) Report 62. The gross tumor volume (GTV) was defined as the radiologically visible primary tumor and involved lymph nodes. The clinical target volume (CTV) for the primary lesion was derived by expanding the primary GTV by 7–8 mm (19), followed by adjustments based on anatomical boundaries. The nodal CTV was created by expanding the nodal GTV by 8 mm and incorporating the draining lymph node regions that were positive prior to chemotherapy. The internal target volume (ITV) was obtained by adjusting the CTV for respiratory motion. The planning target volume (PTV) was defined as the ITV plus an additional 5 mm margin. All treatment plans were uniformly designed to deliver a total dose of 60 Gy in 30 daily fractions. The prescription dose should cover no less than 95% of the PTV. The organs at risk (OARs) in this study included the lungs, heart, esophagus, and spinal cord; for the spinal cord, a 0.5 cm margin was added to form its planning organ-at-risk volume (PRV). The dose limits for OARs were set as follows: Mean lung dose (MLD)< 20 Gy, lung V_20_ (Vx: percentage of volume receiving ≥ x Gy)< 37% (20); Spinal cord PRV maximal dose (D_max_)< 50 Gy (21); Esophagus mean dose (D_mean_)< 34 Gy (22); Mean heart dose (MHD)< 26 Gy, heart V_30_< 46% (23).; FV_10_ (fVx: percentage of functional lung volume receiving ≥ x Gy)<25%, fV_20_<20%, f_MLD_<10 Gy.

### 2.5 Planning design

Treatment planning was designed uniformly using Eclipse TPS (Varian Medical Systems). For every case, both anatomical and functional plans were designed using three techniques: IMRT, hybrid IMRT, and VMAT. Anatomical and functional plans were consistent in terms of prescription dose, target area dose requirements, OAR dose constraints, and weights, with only functional plans additionally including dose constraints for the FL. The PTV was given the highest priority, and the FL dose was minimized under the premise that the OAR dose met clinical requirements. IMRT employed a five-beam arrangement and underwent multiple iterative optimizations until dose-distribution and other metrics met the required standards. Hybrid IMRT combined static fields and intensity-modulated fields, in which static fields delivered four-fifths of the prescription dose, while intensity-modulated fields delivered the remaining one-fifth. VMAT utilized a dual-arc design, with arc selection based on tumor location, and the final treatment plan was optimized iteratively.

### 2.6 Planning evaluation

RT plan evaluation is based on the dose-volume histogram (DVH). For the PTV, the evaluation parameters include D_max_, D_mean_, homogeneity index (HI), and conformity index (CI). The HI is used to assess dose homogeneity within the PTV, with a value closer to 1 indicating a more uniform dose distribution. The calculation formula is shown in Equation 1, where D_5%_ represents the dose received by 5% of the PTV volume and D_95%_ represents the dose received by 95% of the PTV volume.

(1) $$\text{HI}=\frac{{\text{D}}_{5\%}}{{\text{D}}_{95\%}}$$

The CI is used to evaluate the degree of coincidence between the prescription dose isodose and the geometric shape of the PTV, with a value closer to 1 indicating better conformity. The calculation formula is shown in Equation 2, where V_PTV_ denotes the PTV volume, V_PTV_ref_ denotes the PTV volume enclosed by the isodose line of the prescription dose, and V_ref_ denotes the total volume of the irradiated region enclosed by the isodose line of the prescription dose.

(2) $$\text{CI}=\frac{{\text{V}}_{{\text{PTV}}_{\text{ref}}}}{{\text{V}}_{\text{PTV}}}\times \frac{{\text{V}}_{{\text{PTV}}_{\text{ref}}}}{{\text{V}}_{\text{ref}}}$$

For FL and whole lung, evaluate V_5_, V_10_, V_20_, V_30_ and MLD; for the heart, evaluate V_20_, V_30_, V_40_, V_45_ and MHD; for the spinal cord, evaluate D_max_; for the esophagus, evaluate D_max_ and D_mean_.

### 2.7 Statistics

Statistical analysis of the study data was performed using SPSS (IBM SPSS Statistics, USA). For data conforming to a normal distribution, a paired t-test was used; otherwise, the Wilcoxon signed-rank test was applied to compare dosimetric parameters. P values< 0.05 were considered statistically significant.

## 3 Results

### 3.1 Comparison of anatomical and functional plans

**Figure 1:**
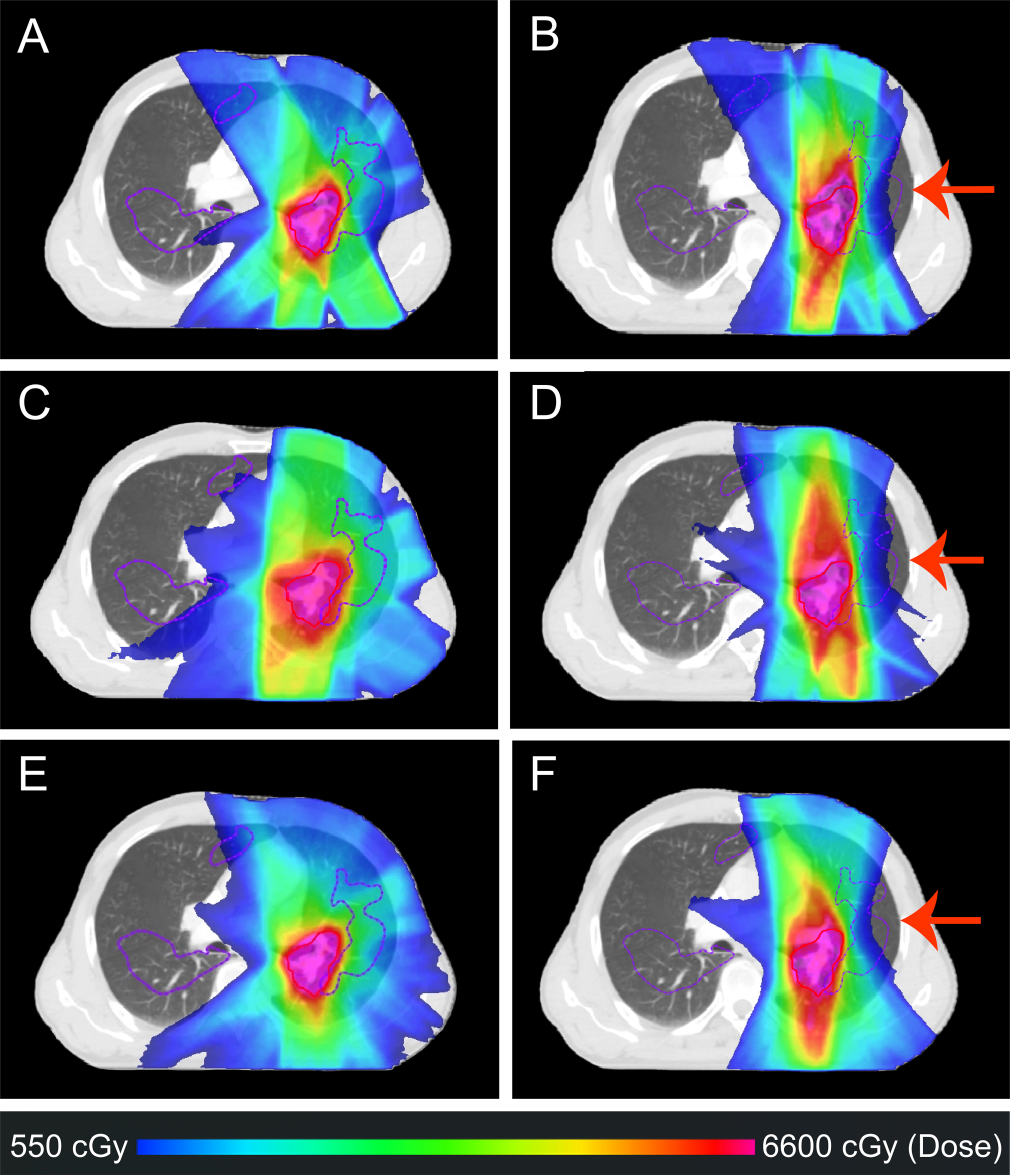
Example isodose distributions of anatomical and functional plans in different RT techniques. The planning target volume (PTV) is highlighted in red and functional lung (FL) regions are in purple. The red arrow indicates the area with the greatest FL preservation in this patient. **(A)** anatomical intensity-modulated radiotherapy (a-IMRT); **(B)** functional IMRT (f-IMRT); **(C)** anatomical hybrid IMRT (a-hybrid IMRT); **(D)** functional hybrid IMRT (f-hybrid IMRT); **(E)** anatomical volumetric modulated arc therapy (a-VMAT); **(F)** functional VMAT (f-VMAT).

**Figure 2:**
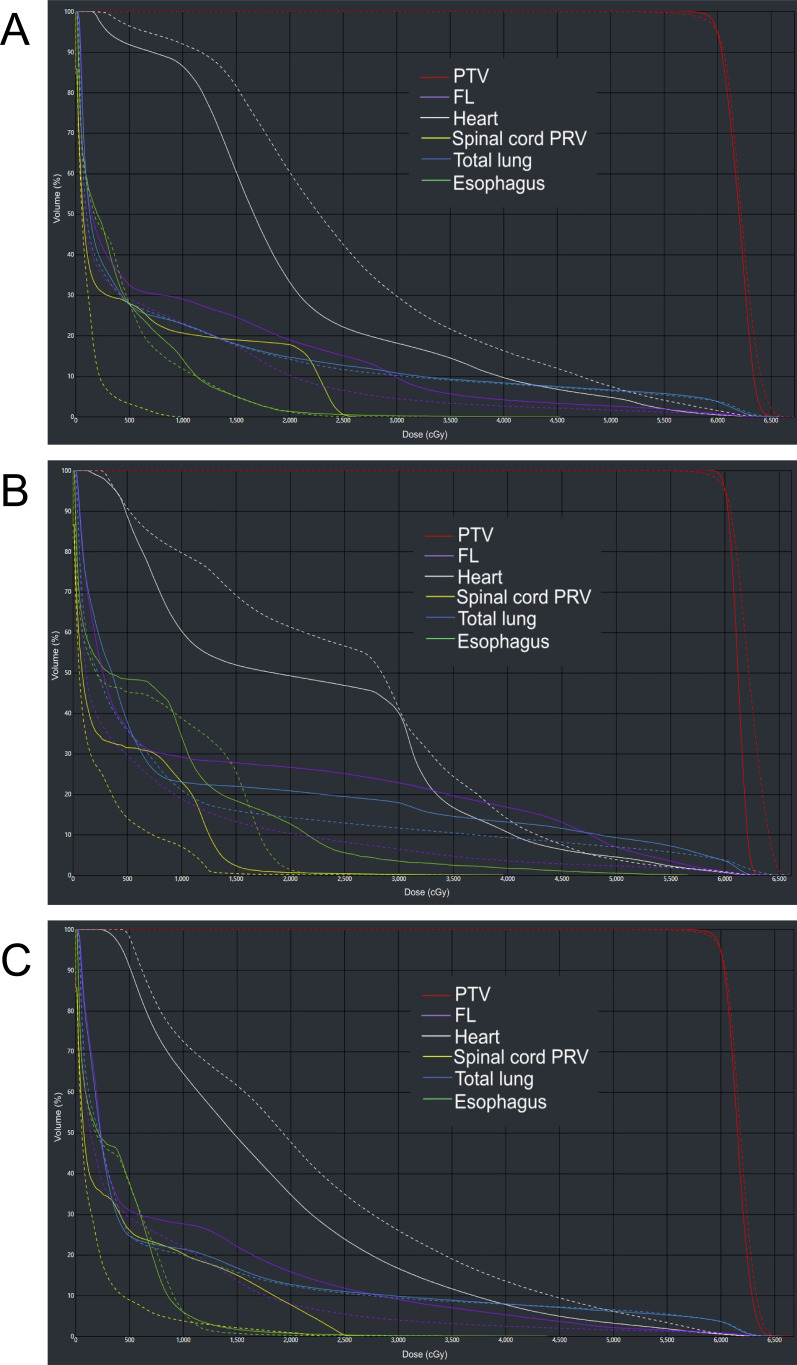
Example dose-volume histograms (DVHs) of anatomical and functional plans in different RT techniques. Anatomical plans are represented by solid lines, and functional plans are represented by dashed lines. The colors corresponding to each structure in the figure are as follows: planning target volume (PTV, red), functional lung (FL, purple), whole lung (blue), heart (white), spinal cord planning organ-at-risk volume (PRV, yellow), and esophagus (green). **(A)** anatomical intensity-modulated radiotherapy (a-IMRT) vs. functional IMRT (f-IMRT); **(B)** anatomical hybrid IMRT (a-hybrid IMRT) vs. functional hybrid IMRT (f-hybrid IMRT); **(C)** anatomical volumetric modulated arc therapy (a-VMAT) vs. functional VMAT (f-VMAT).

For a representative patient, **Figure 1** illustrates the isodose distributions of anatomical and functional plans, with both plan types utilizing IMRT, hybrid IMRT, and VMAT. The red arrows indicate the areas where functional plans provide the best protection for the FL. **Figure 2** shows the DVHs of this patient. The results indicate that, compared with anatomical plans, functional plans reduce the radiation dose to the FL, while doses to OARs satisfy clinical dose constraints. **Table 2** shows the dosimetric comparison between anatomical plans and functional plans. The results showed that regardless of the RT technique used, functional plans reduced the radiation dose to FL compared with anatomical plans. In f-IMRT, functional mean lung dose (fMLD) decreased by 1.45 Gy (*P* < 0.001), while fV_5_, fV_10_, fV_20_, and fV_30_ decreased by 1.91% (*P* = 0.006), 3.62% (*P* = 0.039), 3.56% (*P* < 0.001), and 2.14% (*P* < 0.001), respectively. In f-hybrid IMRT, the corresponding reductions were 2.02 Gy (*P* < 0.001), 2.86% (*P* = 0.006), 2.51% (*P* < 0.001), 3.31% (*P* = 0.016), and 3.83% (*P* = 0.003). In f-VMAT, these parameters decreased by 1.39 Gy (*P* < 0.001), 1.6% (*P* = 0.019), 2.84% (*P* = 0.007), 3.83% (*P* = 0.001), and 3.18% (*P* = 0.002), respectively. Meanwhile, compared with anatomical plans, functional plans showed a decreasing trend in the dosimetric parameters for the total lung, which might be related to the restriction of the FL. The radiation doses to most OARs were higher in functional plans, but none of these differences reached statistical significance. In terms of the PTV metrics, functional plans resulted in an increase in HI compared to anatomical plans. Although the difference was statistically significant, the change was negligible and its clinical significance was limited. Regarding CI, values for functional plans were slightly lower than those for anatomical plans, but the difference was not statistically significant.

**Table 2:** Comparison of dosimetric parameters for anatomical versus functional plans in IMRT, Hybrid IMRT, and VMAT.

Metrics	a-IMRT	f-IMRT	*P* value	a-hybrid IMRT	f-hybrid IMRT	*P* value	a-VMAT	f-VMAT	*P* value
PTV metrics									
D_max_,Gy	66.23 ± 1.48	66.64 ± 1.79	0.042	64.36 ± 0.99	65.49 ± 1.33	<0.001	65.91 ± 1.31	66.30 ± 1.52	0.032
D_mean_,Gy	61.78 ± 0.39	61.87 ± 0.42	0.027	61.32 ± 0.42	61.60 ± 0.46	0.006	61.76 ± 0.44	61.87 ± 0.42	0.020
HI	1.056 ± 0.011	1.059 ± 0.011	0.017	1.043 ± 0.013	1.054 ± 0.015	0.002	1.055 ± 0.013	1.058 ± 0.011	0.039
CI	0.794 ± 0.062	0.784 ± 0.057	0.107	0.691 ± 0.179	0.665 ± 0.147	0.139	0.796 ± 0.074	0.795 ± 0.062	0.961
FL metrics									
fV_5_, %	33.42 ± 18.99	31.51 ± 18.76	0.006	38.87 ± 18.56	36.01 ± 18.15	0.006	31.86 ± 16.48	30.26 ± 16.89	0.019
fV_10_, %	27.75 ± 17.81	24.13 ± 14.52	0.039	31.11 ± 16.36	28.60 ± 17.33	<0.001	26.05 ± 14.08	23.21 ± 12.40	0.007
fV_20_, %	18.13 ± 11.18	14.57 ± 10.22	<0.001	23.43 ± 14.53	20.12 ± 12.27	0.016	18.50 ± 10.45	14.67 ± 10.14	0.001
fV_30_, %	12.50 ± 9.61	10.36 ± 8.75	<0.001	17.54 ± 10.78	13.71 ± 9.73	0.003	13.33 ± 9.65	10.15 ± 9.03	0.002
fMLD, Gy	10.13 ± 5.90	8.68 ± 5.05	<0.001	12.46 ± 6.04	10.44 ± 5.28	<0.001	9.96 ± 5.32	8.57 ± 5.08	<0.001
OAR metrics									
Total lung									
V_5_, %	31.83 ± 9.37	31.59 ± 9.21	0.034	37.18 ± 10.32	36.34 ± 9.77	0.094	31.29 ± 8.89	31.44 ± 8.90	0.525
V_10_, %	25.57 ± 7.79	24.93 ± 8.04	0.023	29.32 ± 8.12	28.01 ± 7.86	0.142	24.95 ± 7.25	24.68 ± 7.56	0.123
V_20_, %	18.29 ± 7.24	17.90 ± 7.14	0.007	21.37 ± 6.71	20.42 ± 6.84	0.066	17.63 ± 6.66	17.47 ± 6.74	0.233
V_30_, %	13.67 ± 5.91	13.46 ± 5.88	0.146	16.48 ± 6.16	15.35 ± 5.82	0.013	13.32 ± 5.60	13.02 ± 5.49	0.087
MLD, Gy	9.96 ± 3.05	9.81 ± 3.11	0.001	11.87 ± 3.35	11.20 ± 3.18	<0.001	9.85 ± 3.02	9.72 ± 3.06	0.020
Heart									
V_20_, %	18.86 ± 12.72	19.88 ± 15.74	0.776	22.10 ± 14.66	22.70 ± 14.99	0.613	17.89 ± 12.08	18.23 ± 13.24	0.691
V_30_, %	12.16 ± 8.68	12.31 ± 9.31	0.435	15.20 ± 11.34	16.12 ± 11.53	0.249	11.67 ± 8.72	12.05 ± 9.21	0.510
V_40_, %	7.32 ± 6.09	7.66 ± 6.51	0.434	10.13 ± 7.71	11.03 ± 8.21	0.195	7.01 ± 5.95	7.37 ± 6.15	0.407
V_45_, %	5.17 ± 4.26	5.52 ± 4.67	0.438	8.23 ± 6.51	8.29 ± 6.54	0.944	4.79 ± 3.94	5.18 ± 4.07	0.170
MHD, Gy	9.90 ± 5.66	10.02 ± 6.40	0.727	11.77 ± 6.48	11.95 ± 6.72	0.618	9.53 ± 5.40	9.65 ± 5.81	0.689
Spinal cord									
D_max_, Gy	36.24 ± 9.77	35.14 ± 11.55	0.711	36.46 ± 10.61	37.54 ± 9.28	0.777	35.36 ± 12.05	34.40 ± 12.31	0.267
Esophagus									
D_max_, Gy	50.67 ± 14.70	49.47 ± 15.25	0.157	51.89 ± 15.67	52.23 ± 14.07	0.528	48.60 ± 16.29	47.98 ± 17.90	0.711
D_mean_, Gy	8.58 ± 6.60	8.48 ± 6.61	0.227	10.54 ± 6.89	10.83 ± 7.12	0.514	7.97 ± 6.91	7.80 ± 6.93	0.112

a-IMRT, anatomical intensity-modulated radiotherapy; a-hybrid IMRT, anatomical hybrid IMRT; a-VMAT, anatomical volumetric modulated arc therapy; f-IMRT, functional intensity-modulated radiotherapy; f-hybrid IMRT, functional hybrid IMRT; f-VMAT, functional volumetric modulated arc therapy; fVx, percentage of functional lung volume receiving ≥ x Gy; PTV, planning target volume; FL, functional lung; OARs, organs at risk; CI, conformability index; HI, homogeneity index; Dmax, maximum dose; Dmean, mean dose; MLD, mean lung dose; MHD, mean heart dose; Vx, percentage of volume receiving ≥ x Gy. Values are expressed as the mean ± standard deviation.

### 3.2 Comparison of functional plans using different RT techniques

To further clarify the differences among these three RT techniques, we conducted a dosimetric comparison of them, detailed in **Table 3**. When comparing f-IMRT with f-VMAT, the two techniques were found to be similar in terms of dosimetric parameters for the PTV and FL, with differences showing no statistical significance. Regarding the dose to OARs, f-VMAT achieved a 0.68 Gy reduction in the esophagus D_mean_ compared with f-IMRT (*P* < 0.001). Compared with f-IMRT and f-VMAT, f-hybrid IMRT showed significant differences. In terms of PTV metrics, f-hybrid IMRT resulted in lower D_max_, D_mean_, and CI(P< 0.05), while no significant difference was found in HI. Regarding the dose to FL, compared with f-IMRT, f-hybrid IMRT increased fV_5_, fV_10_, fV_20_, and fV_30_ by 4.5% (*P* = 0.037), 4.47% (*P* = 0.003), 5.55% (*P* = 0.008), and 3.35% (*P* = 0.002), respectively, and increased fMLD by 1.76 Gy (*P* < 0.001). Compared with f-VMAT, f-hybrid IMRT increased the above metrics by 5.75% (*P* = 0.017), 5.39% (*P* = 0.002), 5.45% (*P* = 0.008), and 3.56% (*P* = 0.004), respectively, and increased fMLD by 1.87 Gy (*P* < 0.001). Regarding OAR exposure, f-hybrid IMRT resulted in higher doses, with statistically significant differences observed for most metrics. Only the spinal cord D_max_ and esophageal D_max_ showed no statistically significant differences (*P* > 0.05).

**Table 3:** Comparison of dosimetric parameters in f-IMRT, f-hybrid IMRT, and f-VMAT.

Metrics				*P* value		
	f-IMRT	f-hybrid IMRT	f-VMAT	f-IMRT vs. f-hybrid IMRT	f-IMRT vs. f-VMAT	f-VMAT vs. f-hybrid IMRT
PTV metrics						
D_max_,Gy	66.64 ± 1.79	65.49 ± 1.33	66.30 ± 1.52	0.003	0.188	0.017
D_mean_,Gy	61.87 ± 0.42	61.60 ± 0.46	61.87 ± 0.42	0.013	0.986	0.009
HI	1.059 ± 0.011	1.054 ± 0.015	1.058 ± 0.011	0.081	0.617	0.227
CI	0.784 ± 0.057	0.665 ± 0.147	0.795 ± 0.062	0.002	0.185	0.001
FL metrics						
fV_5_, %	31.51 ± 18.76	36.01 ± 18.15	30.26 ± 16.89	0.037	0.436	0.017
fV_10_, %	24.13 ± 14.52	28.60 ± 17.33	23.21 ± 12.40	0.003	0.341	0.002
fV_20_, %	14.57 ± 10.22	20.12 ± 12.27	14.67 ± 10.14	0.008	0.854	0.008
fV_30_, %	10.36 ± 8.75	13.71 ± 9.73	10.15 ± 9.03	0.002	0.381	0.004
fMLD, Gy	8.68 ± 5.05	10.44 ± 5.28	8.57 ± 5.08	<0.001	0.424	<0.001
OAR metrics						
Total lung						
V_5_, %	31.59 ± 9.21	36.34 ± 9.77	31.44 ± 8.90	<0.001	0.727	<0.001
V_10_, %	24.93 ± 8.04	28.01 ± 7.86	24.68 ± 7.56	<0.001	0.529	<0.001
V_20_, %	17.90 ± 7.14	20.42 ± 6.84	17.47 ± 6.74	0.003	0.123	<0.001
V_30_, %	13.46 ± 5.88	15.35 ± 5.82	13.02 ± 5.49	0.002	0.017	<0.001
MLD, Gy	9.81 ± 3.11	11.20 ± 3.18	9.72 ± 3.06	<0.001	0.191	<0.001
Heart						
V_20_, %	19.88 ± 15.74	22.70 ± 14.99	18.23 ± 13.24	0.031	0.056	<0.001
V_30_, %	12.31 ± 9.31	16.12 ± 11.53	12.05 ± 9.21	0.004	0.593	0.006
V_40_, %	7.66 ± 6.51	11.03 ± 8.21	7.37 ± 6.15	0.004	0.656	0.005
V_45_, %	5.52 ± 4.67	8.29 ± 6.54	5.18 ± 4.07	0.007	0.445	0.008
MHD, Gy	10.02 ± 6.40	11.95 ± 6.72	9.65 ± 5.81	0.001	0.178	<0.001
Spinal cord						
D_max_, Gy	35.14 ± 11.55	37.54 ± 9.28	34.40 ± 12.31	0.199	0.862	0.372
Esophagus						
D_max_, Gy	49.47 ± 15.25	52.23 ± 14.07	47.98 ± 17.90	0.133	0.492	0.085
D_mean_, Gy	8.48 ± 6.61	10.83 ± 7.12	7.80 ± 6.93	<0.001	<0.001	<0.001

f-IMRT, functional intensity-modulated radiotherapy; f-hybrid IMRT, functional hybrid IMRT; f-VMAT, functional volumetric modulated arc therapy; fVx, percentage of functional lung volume receiving ≥ x Gy; PTV, planning target volume; FL, functional lung; OARs, organs at risk; CI, conformability index; HI, homogeneity index; Dmax, maximum dose; Dmean, mean dose; MLD, mean lung dose; MHD, mean heart dose; Vx, percentage of volume receiving ≥ x Gy. Values are expressed as the mean ± standard deviation.

## 4 Discussion

This study demonstrated the dosimetric feasibility of 4DCT ventilation imaging-guided functional planning in reducing radiation dose to the FL using IMRT, hybrid IMRT, and VMAT. In addition, the study found that f-IMRT and f-VMAT were superior to f-hybrid IMRT, and that f-VMAT further reduced the esophagus D_mean_ compared with f-IMRT.

Currently, there are some studies that explore the dosimetric advantages of 4DCT ventilation imaging-guided functional planning. Studies by Vinogradskiy et al. (24) showed that, in IMRT, compared with anatomical plans, functional plans reduced fMLD by 1.3 Gy (*P* < 0.001), while fV_5_, fV_10_, fV_20_, and fV_30_ decreased by 3.4%, 6.4%, 3.5%, and 1.8%, respectively (*P* < 0.001).Although functional plans increased the spinal cord D_max_ by 1.4 Gy (*P* < 0.001), the dose remained within clinical constraints. Dougherty et al. (14) enrolled 31 patients with lung cancer and designed anatomical and functional plans using VMAT and IMPT, with a significance level of α = 0.002. The results demonstrated that when the dose constraints for target volumes and OARs were met, f-VMAT reduced fV_5_, fV_10_, fV_20_, fV_30_, fV_40_, fV_50_, and f_MLD_ by 2.64% (P = 0.003), 8.88% (P< 0.001), 3.72% (P< 0.001), 2.01% (P< 0.001), 1.26% (P< 0.001), 0.51% (P = 0.006), and 1.56 Gy (P< 0.001), respectively, compared with anatomical plans. For f-IMPT, the corresponding reductions were 3.38%, 4.27%, 4.11%, 2.60%, 1.69%, 0.96%, and 1.51 Gy, with all differences statistically significant (P< 0.001). The results of this study are consistent with the core conclusions of previous research, collectively demonstrating that functional planning exhibits stable FL sparing efficacy across various techniques. In addition, Deng et al. (25) indicated that dose optimization for functional planning should take the degree of tumor regression during RT into account. For patients with significant tumor regression (median regression of 56.2%), improved pulmonary ventilatory function enables tolerance to minor injuries from low-dose irradiation, with priority given to regulating high-dose parameters (> 40 Gy) in highly ventilated lung regions. In contrast, highly ventilated lung regions in patients with poor tumor regression (median regression of 3.5%) are highly sensitive across the entire dose range, requiring strict control of both high−and low−dose parameters. Future functional planning may use tumor regression as a stratification factor to dynamically adjust dose-limiting strategies for highly ventilated lung regions.

Hybrid IMRT, which is a technique of static plus IMRT beams treated concurrently, was initially used for RT of breast cancer. Mayo et al. (26). first applied hybrid IMRT in the treatment of lung cancer. In the treatment plan they developed, static fields delivered two-thirds of the prescribed dose, while intensity-modulated fields delivered the remaining one-third. The results showed that compared with the 9-field IMRT, hybrid IMRT significantly reduced lung V_5_, V_13_, and V_20_, with statistically significant differences (*P* = 0.0005, 0.002, and 0.008, respectively). However, Li et al. (27) reached different conclusions. In their hybrid IMRT plans, static fields delivered four-fifths of the prescribed dose, while intensity-modulated fields delivered the remaining one-fifth. Compared with IMRT, hybrid IMRT resulted in increases of 1.59%, 1.81%, 0.12%, and 4.88% in lung V_5_, V_13_, V_20_, and V_30_, respectively, and MLD increased by 2.33 Gy. Only the difference in V_30_ was statistically significant (*P* = 0.027). In this study, we demonstrated that hybrid IMRT resulted in a higher radiation dose to lung tissue compared with IMRT. This finding is more consistent with the findings of Li et al. but differs from the conclusions of Mayo et al. This discrepancy may be attributed to variations in the dose proportioning between static fields and intensity-modulated beams, as well as the selection of beam incident angles in hybrid IMRT. Future studies should establish unified technical specifications for key parameters such as dose allocation ratio, adopt large-sample study designs, and develop standardized treatment protocols.

In addition, previous studies have compared different RT techniques used in 4DCT ventilation imaging-guided functional planning. Yamamoto et al. (28) found no significant differences between f-IMRT and f-VMAT in the FL dose, PTV metrics, or dose to OARs. However, other studies have demonstrated differences between f-IMRT and f-VMAT. Kimura et al. (29) compared dosimetric parameters between the two techniques in lung cancer patients who also suffered from chronic obstructive pulmonary disease. They defined FL as the total lung volume excluding regions with a CT value of< -860 Hounsfield units. Study findings indicated that, in comparison with f-IMRT, f-VMAT significantly increased fV_5_ by 18.7% (*P* = 0.001), fV_10_ by 8.4% (*P* = 0.045), and fMLD by 0.9 Gy (*P* = 0.002), while reducing fV_20_ by 1.3% (*P* = 0.027). Additionally, f-VMAT reduced CI by 0.64 (*P* = 0.04) and increased esophagus D_mean_ by 3.6 Gy (*P* = 0.003). The results of this study differ from those of previous studies, which showed that f-VMAT reduced the esophagus D_mean_ by 0.68 Gy compared with f-IMRT (*P* < 0.001). Differences in results across studies may be attributed to variations in patient selection, definition of FL, and optimization methods of RT plans. Regarding the clinical benefit of reducing esophageal D_mean_, He et al. (30) confirmed that esophageal D_mean_ was significantly positively correlated with the incidence of grade 1 radiation esophagitis (RE). Within the 0–30 Gy range, the incidence of grade 1 RE increased from 18.34% to 66.35%, with no absolute safe dose threshold, suggesting that a modest reduction in D_mean_ could reduce the risk of grade 1 RE. Therefore, f-VMAT may be considered as a preferable option.

Studies have investigated the association between functional planning and clinical benefits. Dougherty et al. (14) used normal tissue complication probability models to estimate the risk of RP. The results showed that compared with anatomical planning, functional planning resulted in a decrease of 5.7% and 6.2% in Grade 2+ and Grade 3+ RP respectively in intensity modulated proton therapy (IMPT), while the corresponding rates decreased by 2.4% and 3.4% respectively in VMAT. In addition, Vinogradskiy et al. (24) conducted a phase 2 multicenter clinical trial. The results showed that functional planning could reduce the rate of Grade 2+ RP to 14.9% compared with a 25% historical rate. Meanwhile, quality of life (QOL) is the second most critical factor for patients when choosing a treatment regimen. Lombardo et al. (31) first reported QOL outcomes in patients treated with functional planning through a multicenter phase II clinical trial. At 12 months post-treatment, only 29.3% of patients experienced clinically meaningful deterioration in QOL, a rate lower than the 47.8% reported in the RTOG 0617 trial. Poiset et al. (32) further analyzed the results of functional planning and the RTOG 0617, also confirming that functional planning offers superior benefits for improving patients’ QOL. These study results indicate that 4DCT ventilation imaging-guided functional planning hold promising prospects for clinical application.

It should be noted that this study has certain limitations to consider. First, it has a small sample size and uses a single-center study. Second, 4DCT ventilation imaging can only reflect airflow in the lungs and therefore cannot fully encompass lung function. Moreover, some patients may have a ventilation-perfusion mismatch (33), which could impair its ability to accurately reflect the condition of the lung tissue. Third, there is no unified consensus on the setting of FL thresholds, and different researchers adopt different standards. More studies are needed in the future to explore the optimal definition method of FL. Finally, this study mainly compared the dosimetric differences between different RT techniques and did not conduct application verification in clinical practice. Its true clinical value still needs to be further confirmed through subsequent studies.

## 5 Conclusion

In conclusion, this study demonstrates that 4DCT ventilation imaging-guided functional planning can reduce the radiation dose to the FL while preserving target dose coverage and meeting dose constraints for OARs. In addition, the analysis of the optimal RT technique for functional planning indicates that routine use of f-hybrid IMRT may not be favorable. Compared with f-IMRT, f-VMAT offers potential clinical advantages in esophageal sparing and may serve as the preferred technique.

## Data Availability

The raw data supporting the conclusions of this article will be made available by the authors, without undue reservation.
